# Evaluation of the utility of different laboratory test-related sarcopenia indices as predictors of lung cancer mortality

**DOI:** 10.1186/s12877-025-05951-4

**Published:** 2025-05-23

**Authors:** Xiaoyan Chen, Shuyue Luo, Lisha Hou, Ming Yang, Qiukui Hao

**Affiliations:** 1 Department of Geriatric, The Zigong Affliated Hospital, SouthwestMedical University, Zigong, Sichuan China; 2 West China Hospital, National Clinical Research Center for Geriatrics, Sichuan University, Chengdu, Sichuan Province China

**Keywords:** Sarcopenia index, Prognosis, Older patients, Lung cancer

## Abstract

**Objectives:**

We evaluated the utility of routine laboratory test-related sarcopenia indices as predictors of mortality in older patients with primary lung cancer undergoing the first chemotherapy course.

**Design:**

Retrospective cohort study.

**Setting:**

West China Hospital, Chengdu, China.

**Participants:**

This study enrolled primary lung cancer patients ≥ 60 years of age undergoing their first chemotherapy course.

**Measurements:**

Data on individual patients were obtained from the medical records, while information on survival outcomes was gathered through telephone-based follow-up or local government databases. Using available routine hematological and biochemical test results, this study calculated three sarcopenia-related indices for each patient. These indices included the AST/ALT ratio, neutrophil-to-lymphocyte ratio (NLR), and platelet-to-lymphocyte ratio (PLR). We assessed the relationships between these indices and death using Cox proportional hazards models.

**Results:**

The study included 926 primary lung cancer patients (71.5% male; median age: 65 years) who underwent their first course of chemotherapy. During the follow-up period (median: 28 months), 563 patients (60.8%) died. In the overall population, there was a significantly higher likelihood of all-cause mortality in patients with an NLR ≥ 2.88 (HR = 1.60, 95% CI = 1.36–1.90, *P* < 0.001) or a PLR ≥ 125.11 (HR = 1.39, 95% CI = 1.17–1.64, *P* < 0.001) compared to those with values below these thresholds. However, after adjustment for potential confounding factors, no association was found between NLR or PLR and mortality. After stratification by sex, it was found that both NLR and PLR values were associated with an increased risk of mortality among women (NLR: HR = 2.1, *P* < 0.001; PLR: HR = 2.42, *P* < 0.001).

**Conclusions:**

NLR and PLR, are indicators of sarcopenia and can be easily derived from routine laboratory testing data. These indices can significantly predict mortality in older female patients with primary lung cancer at the start of chemotherapy. Therefore, there is potential practical value in using these indices for assessing patient risk prior to chemotherapy.

**Supplementary Information:**

The online version contains supplementary material available at 10.1186/s12877-025-05951-4.

## Introduction

Lung cancer remains the most commonly diagnosed malignancy worldwide and a leading cause of cancer-related mortality, particularly among older adults who exhibit a significantly higher risk [1]. In many cases, the disease is diagnosed at an intermediate or advanced stage in this group. The increasingly advanced interventional strategies can provide these patients with opportunities for chemotherapy, radiotherapy, and other therapeutic approaches [2]. Even with these interventions, however, the overall survival (OS) rates in patients with lung cancer remain limited. This highlights a critical need to identify mortality-related risk factors to facilitate a more tailored and effective management of lung cancer.

Sarcopenia, a clinically important age-related syndrome, is characterized by a progressive decline in physical performance, muscle strength, and skeletal muscle mass [3]. Sarcopenia is common among older adults throughout the world and is associated with numerous adverse health outcomes, including an increased risk of falls [4], bone fractures [4], functional disability [5], and mortality [6]. Recently, this condition has emerged as a critical area of focus in oncology, as it has been found to be predictive of adverse outcomes and death among cancer patients [6]. Specifically, cancer patients undergoing chemotherapy and/or radiotherapy who also suffer from sarcopenia are more likely to have poorer outcomes and higher mortality [6].

Regular assessment of sarcopenia status in accordance with established clinical guidelines presents challenges due to the need for specialized analytical strategies such as CT, MRI, BIA, and DEXA. These methods are not only costly but also require a level of expertise that adds to the workload of clinical staff [7]. Early diagnosis of individuals with sarcopenia is crucial, as it enables timely intervention that can help prevent its progression potentially leading to functional disability [8].

For these reasons, researchers have explored simpler alternative indices to evaluate sarcopenia status, such as the AST/ALT ratio [9], the neutrophil-to-lymphocyte ratio (NLR) [10], and the platelet-to-lymphocyte ratio (PLR) [11]. Each of these indices has shown promise in the effective detection of sarcopenia. However, studies examining the correlations between these indices and all-cause mortality in older patients with primary lung cancer undergoing their first chemotherapy course remain limited. This study aimed to investigate the utility of three different sarcopenia indices in predicting all-cause death among older patients with primary lung cancer.

## Methods

### Study design and participants

This single-center retrospective study enrolled patients aged 60 years and over who had pathologically confirmed primary lung cancer of any type. The patients underwent their first course of chemotherapy at West China Hospital, Sichuan University, from January 2010 through December 2017. Patients were excluded if their medical data were missing or they lacked test results from routine hematological or blood biochemistry analyses. Two investigators independently obtained baseline patient characteristics and health-related data through an anonymized electronic medical records system.

### Ethical oversight

This study was conducted retrospectively using patient medical records. All relevant data were anonymized by the Health Informatics Center, which was responsible for supervising the study protocol. Throughout the research, the investigators ensured that all data remained confidential and that all analyses were conducted according to the Declaration of Helsinki. Given the anonymized and retrospective nature of this study, the requirement for patient consent was waived. The Research Ethics Committee of West China Hospital, Sichuan University, approved this study (No. 2018–94).

### Sarcopenia index analyses

To assess the sarcopenia status of patients at baseline, sarcopenia indices were computed using a standard approach. Specifically, three sarcopenia indices were analyzed, each derivable from routine hematological and blood biochemistry test results: the AST/ALT ratio, the neutrophil-to-lymphocyte ratio (NLR), and the platelet-to-lymphocyte ratio (PLR). These variables were measured using fasting blood samples collected prior to the first round of chemotherapy for enrolled patients. The cut-off values for these indices were established based on previous studies with an AST/ALT threshold of 1.35 [12], an NLR threshold of 2.88 [10], and a PLR threshold of 125.11 [13].

### All-cause mortality

Mortality-related data for study participants were initially obtained through a review of local government death registries. In cases where these databases lacked the necessary information, telephone interviews were conducted. The collected mortality data included survival status and the date of death, with a cut-off date for data collection set at April 1, 2018. The median follow-up length was 28 months, and OS was measured from the start of treatment to the occurrence of all-cause death or the last follow-up.

### Covariates

Baseline patient data prior to the initiation of chemotherapy were collected retrospectively, including age, sex, marital status, occupation, health insurance coverage, body mass index (BMI), smoking and drinking status. A variety of tumor-related parameters were also recorded, including histology, clinical stage, and presence of metastasis, along with details regarding the chemotherapy regimens, radiotherapy, and any lung cancer-related surgeries. Patients were categorized based on their smoking history into current smokers, former smokers, and non-smokers, and their total pack-years of cigarette consumption were also assessed. Any adverse reactions experienced by patients during chemotherapy were also recorded.

### Statistical analysis

We conducted statistical analyses using SPSS v24.0 (IBM Corp., NY, USA). A two-sided *P*-value of less than 0.05 was considered statistically significant. Data are presented as numbers (percentages) or means ± standard deviation or median(q25,q75),as appropriate. The Mann-Whitney U, Pearson’s chi-square, and Fisher’s exact tests were utilized for comparisons between groups. Binary logistic regression was utilized to assess the associations between each sarcopenia index and short-term outcomes. Kaplan-Meier curves with log-rank tests were utilized to assess survival. Cox proportional hazards models were used to evaluate the impacts of the sarcopenia indices on all-cause mortality. Odds ratios (ORs) for logistic regression and hazard ratios(HRs) for Cox models,along with95% confidence intervals (CIs) were calculated using an unadjusted model and a model adjusted for age, sex, smoking history, BMI, tissue type, clinical stage, radiotherapy, chemotherapy regimen, surgery, and metastasis.

## Results

### Patient characteristics

Among the 1,263 individuals initially enrolled in this study, 337 were ultimately excluded due to missing medical records or laboratory data. The analysis included the remaining 926 primary lung cancer patients (71.5% male; median age: 65 years) who underwent their first course of chemotherapy. Mortality was found to be significantly higher among males compared to females (66.16% vs. 47.35%, *P* < 0.001). Those who did not survive showed higher rates of smoking than survivors. No statistically significant differences were observed between the groups regarding other conditions such as diabetes, hypertension, and coronary heart disease(CHD).

Adenocarcinoma was the most prevalent histological type among the participants (71.0%), followed by squamous cell carcinoma (15.0%) and small cell carcinoma (14.0%). A majority of these individuals received combination chemotherapy treatment. Compared to survivors, metastatic and advanced disease was more prevalent among individuals who had died (*P* < 0.001). A greater proportion of lung cancer survivors underwent surgery (69.26%) compared to decedents (30.74%) (*p* < 0.001).

Both the NLR (*P* < 0.001) and PLR (*P* = 0.005) were significantly higher among deceased individuals relative to survivors; however, this was not the case for the AST/ALT ratios (*P* = 0.25). Therefore, the AST/ALT values were excluded from subsequent survival analyses (Table 1). Although some patients experienced adverse reactions while undergoing chemotherapeutic treatment, no statistically significant differences were observed in the incidence of these reactions between survivors and non-survivors (Table 2). We also found that there was no significant difference in sarcopenia indicators between the group without adverse reactions after chemotherapy and the group with adverse reactions after chemotherapy (Table 1S).

**Table 1:** According to the general characteristics of the death distribution

Characteristics	Non-death *n* = 363	Death *n* = 563	*P*
**Age (years)**,** median(p25, p75)**	64(62, 69)	65(62, 69)	**0.048**
**Sex**,** n(%)**			**<0.001**
male	224(33.84)	438(66.16)	
female	139(52.65)	125(47.35)	
**Smoking history**,** n(%)**			**<0.001**
no	175(48.88)	183(51.12)	
former smoker	108(38.99)	169(61.01)	
current smoker	80(27.49)	211(72.51)	
**Hypertension**,** n(%)**			0.07
no	264(37.55)	439(62.45)	
yes	96(44.44)	120(55.56)	
**CHD**,** n(%)**			0.394
no	349(38.95)	547(61.05)	
yes	14(46.67)	16(53.33)	
**COPD**,** n(%)**			0.131
no	318(40.2)	473(59.8)	
yes	45(33.33)	90(66.67)	
**Diabetes**,** n(%)**			0.473
no	314(38.77)	496(61.23)	
yes	49(42.24)	67(57.76)	
**Tissue type**,** n(%)**			**<0.001**
adenocarcinoma	288(43.84)	369(56.16)	
squamous cell carcinoma	46(33.09)	93(66.91)	
small cell carcinoma	29(22.31)	101(77.69)	
**Clinical stage**,** n(%)**			**<0.001**
I-II	114(80.28)	28(19.72)	
III-IV	249(31.76)	535(68.24)	
**Regimen of Chemotherapy**,** n (%)**			**0.01**
**single**	21(25.93)	60(74.07)	
**combination**	342(40.47)	503(59.53)	
**Radiation therapy**,** n(%)**			**<0.001**
no	291(44.84)	358(55.16)	
yes	72(25.99)	205(74.01)	
**Metastasis**,** n(%)**			**<0.001**
no	97(80.17)	24(19.83)	
yes	266(33.08)	538(66.92)	
**Surgery**			**<0.001**
no	176(26.87)	479(73.13)	
yes	187(69.26)	83(30.74)	
**BMI**,** kg/m**^**2**^, **mean(SD)**	22.89(2.7)	22.45(3.21)	**0.029**
**ALB level, g/l**	40.8(38.3, 42.8)	38.7(35.6, 41.5)	**<0.001**
**AST/ALT**,** n(%)**			0.25
<1.35	263(40.4)	388(59.6)	
≥ 1.35	100(36.36)	175(63.64)	
**NLR**,** n(%)**			**<0.001**
<2.88	211(48.06)	228(51.94)	
≥ 2.88	152(31.21)	335(68.79)	
**PLR**,** n(%)**			**0.005**
<125.11	179(44.31)	225(55.69)	
≥ 125.11	184(35.25)	338(64.75)	

Note: CHD, Coronary heart disease;

COPD, Chronic obstructive pulmonary disease;

BMI, Body mass index;

ALB, Albumin;

AST/ALT,,aspartate aminotransferase to alanine aminotransferase ratio;

NLR, neutrophil–lymphocyte ratio;

PLR, platelet–lymphocyte ratio;

**Table 2:** Relationship between adverse reactions and mortality

Characteristics	Non-death *n* = 363	Death *n* = 563	χ2/T/Z	*P*
**Bone marrow suppression**,** n(%)**			1.186	0.276
no	303(40.03)	454(59.97)		
yes	60(35.5)	109(64.5)		
**Digestive reactions**,** n(%)**			1.498	0.221
no	338(38.72)	535(61.28)		
yes	25(47.17)	28(52.83)		
**Liver function impairment**,** n(%)**			0.597	0.44
no	335(38.86)	527(61.14)		
yes	28(43.75)	36(56.25)		
**All infection**			1.775	0.183
no	339(39.84)	512(60.16)		
yes	24(32)	51(68)		

### Evaluation of survival status following chemotherapy based on patient sarcopenia indices

**Fig. 1:**
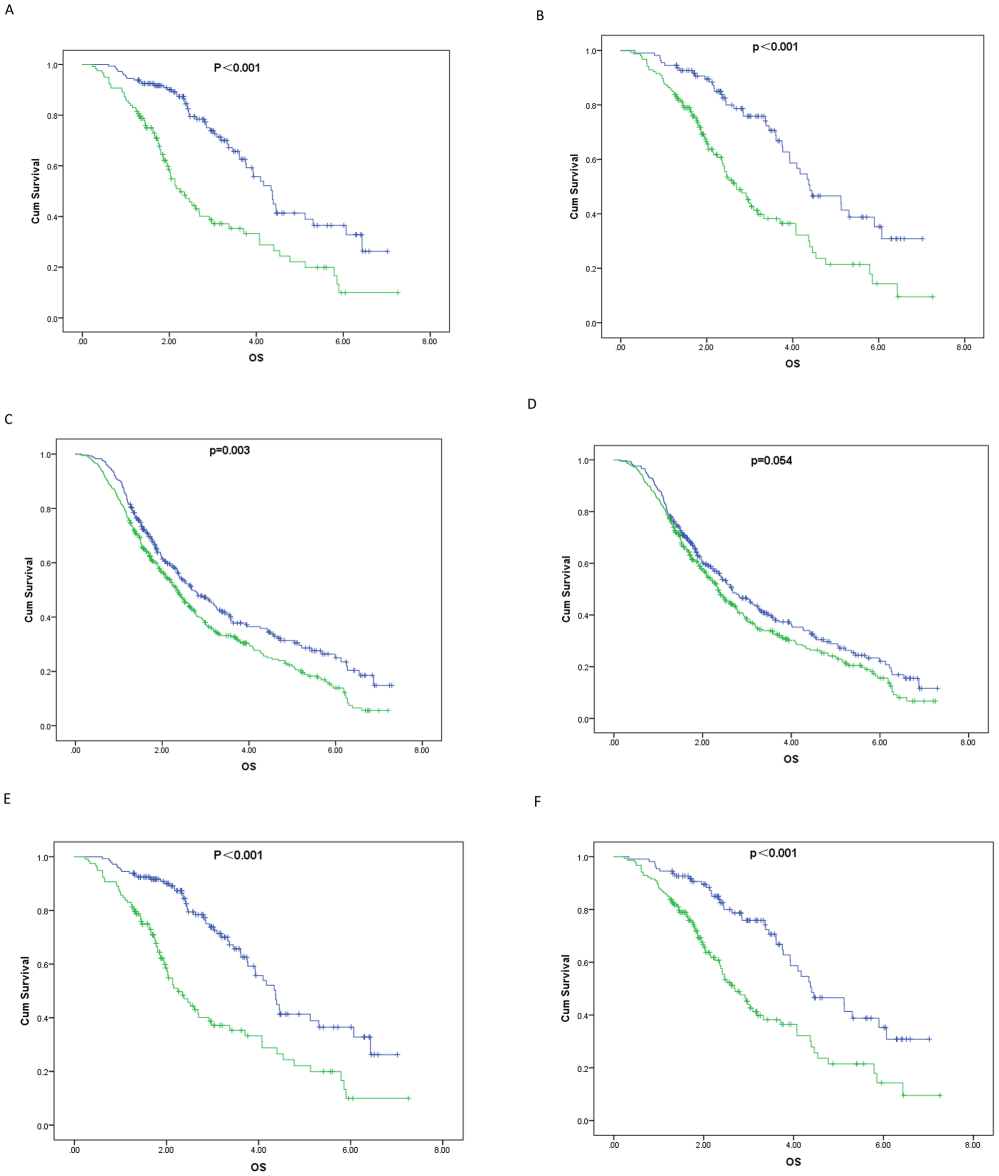
Survival curves with primary lung cancer receiving chemotherapy according to baseline sarcopenia index. Note(years): **A**, NLR(toal); OS(4.527 vs. 3.087);blue, NLR<2.88, green, NLR ≥ 2.88; **B**, PLR(toal); OS(4.65 vs.3.302);blue, PLR<125.11, green, PLR ≥ 125.11; **C**, NLR(male); OS(3.541 vs.2.958);blue, NLR<2.88, green, NLR ≥ 2.88; **D**, PLR(male); OS(3.416 vs. 3.038);blue, PLR<125.11, green, PLR ≥ 125.11; **E**, NLR(female); OS(4.527 vs.3.087);blue, NLR<2.88, green, NLR ≥ 2.88; **F**, PLR(female); OS(4.65 vs. 3.302);blue, PLR<125.11, green, PLR ≥ 125.11;

Of the 926 patients analyzed, 563 died during the follow-up period. Among those who died, 335 (68.79%) had an NLR of 2.88 or greater, and 338 (64.75%) had a PLR of 125.11 or greater. Figure 1 shows the survival curves for the overall cohort and for males and females stratified by the NLR and PLR thresholds. An NLR ≥ 2.88 was associated with significantly poorer survival in the overall cohort and in both sexes (log-rank *P* < 0.001). While males did not show significantly different survival based on PLR ≥ 125.11(*P* = 0.054), both the overall cohort and the female group showed significantly worse survival with PLR ≥ 125.11 (log-rank *P* < 0.001).

In the unadjusted analysis, both an NLR of 2.88 or greater (HR = 1.60, 95% CI = 1.36–1.90, *P* < 0.001) and a PLR of 125.11 or greater (HR = 1.39, 95% CI = 1.17–1.64, *P* < 0.001) were significantly associated with an increased risk of all-cause mortality. However, after adjusting for age, sex, smoking history, BMI, tissue type, clinical stage, chemotherapy regimen, radiotherapy, metastasis, surgery, and ALB level, the association between NLR or PLR and OS was not significant (Table 3).

**Table 3:** Association between sarcopenia index and mortality

Sarcopenia index		Unadjusted		Adjusted	
		*P*-value	HR (95% CI)	*P*-value	HR (95% CI)
**Total**					
	**NLR**				
	<2.88	-	1	-	1
	≥ 2.88	**<0.001**	1.60(1.36–1.9)	0.932	1.01(0.84–1.21)
	**PLR**				
	<125.11	-	1	-	1
	≥ 125.11	**<0.001**	1.39(1.17–1.64)	0.348	0.92(0.76–1.1)
**Male**					
	**NLR**				
	<2.88	-	1	-	1
	≥ 2.88	**0.004**	1.33(1.1–1.61)	0.133	0.85(0.69–1.05)
	**PLR**				
	<125.11	-	1	-	1
	≥ 125.11	0.054	1.21(1-1.46)	0.416	1.09(0.89–1.34)
**Female**					
	**NLR**				
	<2.88	-	1	-	1
	≥ 2.88	**<0.001**	2.52(1.86–3.61)	**<0.001**	2.1(1.42–3.09)
	**PLR**				
	<125.11	-	1	-	1
	≥ 125.11	**<0.001**	2.29(1.57–3.34)	**<0.001**	2.42(1.59–3.68)

Note:

Model 1, Unadjusted model;

Model2, Adjusted for age, sex, smoking history, tissue type, clinical stage, regimen of chemotherapy, radiation therapy, metastasis, surgery, BMI, ALB level

In males, an NLR ≥ 2.88 was significantly associated with poor OS in the unadjusted model (HR = 1.33, 95% CI = 1.1–1.61, *P* = 0.004); however, this association was not statistically significant after adjustment for the above potential confounding factors (HR = 0.85, 95% CI = 0.69–1.05, *P* = 0.133). In males, there was also no significant association between PLR ≥ 125.11 and OS (non-adjusted model: HR = 1.21, 95% CI = 1-1.46, *P* = 0.054; adjusted model: HR = 1.09, 95% CI = 0.89–1.34, *P* = 0.416; Table 3).

In females, both an NLR ≥ 2.88 and a PLR ≥ 125.11 were significantly associated with poor OS, even after adjustment for potential confounding variables (NLR: HR [non-adjusted model] = 2.52, 95% CI = 1.86–3.61, *P* < 0.001; HR [adjusted model] = 2.1, 95% CI = 1.42–3.09, *P* < 0.001; PLR: HR [non-adjusted model] = 2.29, 95% CI = 1.57–3.34, *P* < 0.001; HR [adjusted model] = 2.42, 95% CI = 1.59–3.68, *P* < 0.001; Table 3).

## Discussion

This study investigated the predictive value of sarcopenia indices, specifically, the NLR and PLR, for all-cause mortality in 926 patients aged 60 and over with primary lung cancer undergoing their first cycle of chemotherapy. It was found that both NLR and PLR were associated with OS, suggesting their potential as accessible risk-assessing tools for older patients with lung cancer in clinical practice. Laboratory-based indicators (such as NLR and PLR) require only routine blood tests, eliminating the need for additional imaging analyses or specialized equipment. This makes them particularly advantageous in resource-limited settings, healthcare institutions where CT scans are not readily available, or for community populations and patients. Furthermore, for patients who have already undergone CT scans, laboratory indicators can be used in conjunction with imaging metrics to provide a more comprehensive dynamic follow-up assessment of prognosis.

While previous studies have observed an association between NLR values and all-cause mortality in patients with non-small cell lung cancer [14, 15], we observed this association only in female patients. PLR, similarly, was associated with increased mortality risk in this female subgroup. The potential reasons for the differences between males and females may include the following: (1) Biological mechanisms underlying sex differences, as there are significant differences in hormone levels (such as estrogen and androgen) between males and females. Estrogen has anti-inflammatory effects, which may enhance female sensitivity to inflammatory markers (such as NLR and PLR). In contrast, male sex may be associated with weaker NLR and PLR predictive ability due to the lack of estrogen [16]. (2) Sex differences in immune responses: studies have shown that females generally have more active immune systems compared to males, indicating that inflammatory markers may be more reflective of disease status or prognosis in women [17]. (3) For chemotherapy tolerance, studies indicate that females may experience higher toxicity rates and different therapeutic outcomes compared to males in lung cancer [18].

Several biological mechanisms may explain the association between NLR or PLR and mortality. Neutrophils are essential components of the innate immune system involved in counteracting infections and mediating inflammatory activity. The specific roles that neutrophils play in cancer, however, remain somewhat controversial, potentially owing to their plasticity and the effects of the tumor microenvironment. Both neutrophils and platelets ultimately interact with the tumor microenvironment, influencing immune responses and promoting immune evasion and tumor progression [19, 20]. These neutrophils and platelets release cytokines and chemokines, such as TGF-β, VEGF, IL-6, and IL-8 [19]. Reductions in lymphocyte counts also tend to be indicative of the impairment of cellular immunity and the corresponding disruption of the immunological landscape. Moreover, neutrophils can release inflammatory mediators that suppress T-cell functions and alter tumor angiogenesis [21, 22]. Coffelt et al. proposed that targeting a novel immune axis consisting of γδ T cells, IL-17, and neutrophils may contribute to a greater risk of metastasis [23]. These complex interactions may clarify how these indices are related to tumor progression and immune escape.

Older patients with cancer show a high degree of heterogeneity due to the presence of various comorbidities and differences in the manifestation of aging-related changes [24, 25]. Personalized treatment strategies should be employed to gauge both the toxicity-related risks and survival prospects of older cancer patients undergoing treatment [26, 27]. Sarcopenia, which is associated with both the NLR and PLR [28, 29], can contribute to a heightened inflammatory state, further impacting these ratios [30]. This concurrent inflammation can also exacerbate muscle loss, initiating a vicious cycle that negatively affects physical function and treatment outcomes [31]. Therefore, monitoring the NLR and PLR can be useful in assessing the health status of cancer patients and gauging the progression of sarcopenia [6]. Notably, the NLR is not only a sarcopenia index but also forms part of the Royal Marsden Hospital (RMH) score [32]. Furthermore, the metabolic indicators in the RMH score, such as serum albumin, are closely related to sarcopenia, as sarcopenia itself is a syndrome associated with metabolic dysregulation and malnutrition. Therefore, both the NLR and PLR may influence cancer prognosis not only through inflammatory pathways but also *via* metabolic interactions with sarcopenia, further impacting patient survival. The findings of this study provide new insights into the multifaceted roles of the NLR and PLR in cancer prognosis. Future research could explore the relationship between sarcopenia and the RMH score, such as whether patients with sarcopenia are more likely to have higher RMH scores and whether this association is independent of other known prognostic factors.

This study has several limitations. First, the single-center and retrospective design, along with the limited sample size, may have contributed to potential selection bias. Furthermore, several indicators were lacking from the analysis, such as systemic inflammation and Eastern Cooperative Oncology Group scale scores which could not be compensated for due to the retrospective analysis. Second, the study cohort was restricted to individuals of Chinese ethnicity, limiting the generalizability of the results. Third, as we were unable to obtain chest or lumbar spine CT/MRI scans for further analysis. Further exploration in this population should be conducted in future research. Fourth, since analysis of CT images was not possible in this study, the albumin-myosteatosis gauge (AMG) score could not be calculated. The AMG score is typically based on imaging features and reflects the metabolic activity of tumors. Future research could combine imaging indicators (such as AMG) with blood biomarkers (such as NLR and PLR) to more comprehensively evaluate the prognosis of lung cancer patients. Fifth, due to the limited sample size in our study, the relationship between sarcopenia indices (such as NLR and PLR) and survival could not be analyzed further by stratifying patients according to cancer stage. However, this aspect could be explored in larger population-based studies. Sixth, we selected lung cancer patients diagnosed between 2010 and 2017 for analysis. We acknowledge that the widespread use of immunotherapy in recent years may alter the inflammatory status and prognostic patterns of patients, thereby potentially affecting the predictive value of the sarcopenia indices. Future studies are needed to further verify our findings in patient populations that include those receiving immunotherapy.

## Conclusion

Both the NLR and PLR, which can be calculated based on routinely gathered laboratory data, can predict an increased mortality risk among older female lung cancer patients starting chemotherapeutic treatment. The findings highlight the potential role of these indices as accessible and valuable tools for risk assessment before chemotherapy in this population.

## Electronic supplementary material

Below is the link to the electronic supplementary material.

Supplementary Material 1

## Data Availability

If the request is reasonable, the original data can be obtained. Please contact the corresponding author, Professor Hao Qiukui, at haoqiukui@gmail.com.

## Abbreviations

NLR: Neutrophil-to-lymphocyte ratio
PLR: Platelet-to-lymphocyte ratio
OS: Overall survival
BMI: Body mass index
OR: Odds ratio
CI: Confidence interval
RMH: Royal marsden hospital
AMG: Albumin-myosteatosis gauge
