# Supplementary material for: Evaluation of the utility of different laboratory test-related sarcopenia indices as predictors of lung cancer mortality
Source: BMC Geriatr. 2025 May 23;25:367. doi: 10.1186/s12877-025-05951-4 (PMC12100790; doi:10.1186/s12877-025-05951-4)
Supplement: Supplementary file 1 — Supplementary Material 1 [file 12877_2025_5951_MOESM1_ESM.docx]

**Table 1S Relationship between sarcopenia indices and adverse reactions**

| **Characteristics** | **Non-bone marrow suppression**  **n=757** | **Bone marrow suppression**  **n=169** | **χ2/T/Z** | ***P*** |
| --- | --- | --- | --- | --- |
| **AST/ALT，n（%）** |  |  | 0.049 | 0.825 |
| ＜1.35 | 531(81.57) | 120（18.43） |  |  |
| ≥1.35 | 226（82.18） | 49（17.82） |  |  |
| **NLR,n(%)** |  |  | 1.803 | 0.179 |
| ＜2.88 | 351（79.95） | 88（20.05） |  |  |
| ≥2.88 | 406（83.37） | 81（16.63） |  |  |
| **PLR,n(%)** |  |  | 1.156 | 0.282 |
| ＜125.11 | 324（80.2） | 80（19.8） |  |  |
| ≥125.11 | 433（82.95） | 89（17.05） |  |  |

| **Characteristics** | **Non-digestive reactions**  **n=873** | **Digestive reactions**  **n=53** | **χ2/T/Z** | ***P*** |
| --- | --- | --- | --- | --- |
| **AST/ALT，n（%）** |  |  | 0.72 | 0.396 |
| ＜1.35 | 611（93.86） | 40（6.14） |  |  |
| ≥1.35 | 262（95.27） | 13（4.73） |  |  |
| **NLR,n(%)** |  |  | 1.204 | 0.272 |
| ＜2.88 | 410（93.39） | 29（6.61） |  |  |
| ≥2.88 | 463（95.07） | 24（4.93） |  |  |
| **PLR,n(%)** |  |  | 1.223 | 0.269 |
| ＜125.11 | 377（93.32） | 27（6.68） |  |  |
| ≥125.11 | 496（95.02） | 26（4.98） |  |  |

| **Characteristics** | **Non-All infection**  **n=851** | **All infection**  **n=75** | **χ2/T/Z** | ***P*** |
| --- | --- | --- | --- | --- |
| **AST/ALT，n（%）** |  |  | 0.113 | 0.737 |
| ＜1.35 | 597（91.71） | 54（8.29） |  |  |
| ≥1.35 | 254（92.36） | 21（7.64） |  |  |
| **NLR,n(%)** |  |  | 2.501 | 0.114 |
| ＜2.88 | 410（93.39） | 29（6.61） |  |  |
| ≥2.88 | 441（90.55） | 46（9.45） |  |  |
| **PLR,n(%)** |  |  | 1.931 | 0.165 |
| ＜125.11 | 377（93.32） | 27（6.68） |  |  |
| ≥125.11 | 474（90.8） | 48（9.2） |  |  |
